# Combining fluorescence and bioluminescence microscopy

**DOI:** 10.1002/jemt.22529

**Published:** 2015-06-19

**Authors:** Kazuhito Goda, Yoko Hatta‐Ohashi, Ryutaro Akiyoshi, Takashi Sugiyama, Ikuko Sakai, Takeo Takahashi, Hirobumi Suzuki

**Affiliations:** ^1^ Corporate Research and Development Center, Olympus CorporationHachiojiTokyo192‐8512Japan

**Keywords:** luciferin, PKC, NF‐κB, single‐cell analysis

## Abstract

Bioluminescence microscopy has revealed that gene expression in individual cells can respond differently to the same stimulus. To understand this phenomenon, it is important to sequentially observe the series of events from cellular signal transduction to gene expression regulated by specific transcription factors derived from signaling cascades in individual cells. However, these processes have been separately analyzed with fluorescence and bioluminescence microscopy. Furthermore, in culture medium, the background fluorescence of luciferin—a substrate of luciferase in promoter assays of gene expression in cultured cells—confounds the simultaneous observation of fluorescence and bioluminescence. Therefore, we optimized conditions for optical filter sets based on spectral properties and the luciferin concentration based on cell permeability for fluorescence observation combined with bioluminescence microscopy. An excitation and emission filter set (492–506 nm and 524–578 nm) was suitable for green fluorescent protein and yellow fluorescent protein imaging of cells, and >100 μM luciferin was acceptable in culture medium based on kinetic constants and the estimated intracellular concentration. Using these parameters, we present an example of sequential fluorescence and bioluminescence microscopic observation of signal transduction (translocation of protein kinase C alpha from the cytoplasm to the plasma membrane) coupled with activation of gene expression by nuclear factor of kappa light polypeptide B in individual cells and show that the gene expression response is not completely concordant with upstream signaling following stimulation with phorbol‐12‐myristate‐13‐acetate. Our technique is a powerful imaging tool for analysis of heterogeneous gene expression together with upstream signaling in live single cells. *Microsc. Res. Tech. 78:715–722, 2015*. © 2015 Wiley Periodicals, Inc.

## INTRODUCTION

Gene expression and its regulation are fundamental cellular processes, and techniques to measure transcriptional output are essential for studies of cell proliferation and differentiation. In particular, owing to the sensitivity, wide dynamic range, and assay convenience, the firefly luciferase gene is often used as a reporter gene to investigate gene promoter activity as determined by detection of bioluminescence emission from cells in the presence of the substrate luciferin (Alam and Cook, 1990; Brasier et al., 1989; de Wet et al., 1987). With this technique, bioluminescence is measured in large populations of cells using a photon‐counting luminometer because light emission from the cells is too weak to capture images of the cells with conventional charge‐coupled device (CCD) cameras.

Bioluminescence imaging of promoter activity in single cells has been performed using microscopes equipped with ultra‐low‐light imaging cameras, such as liquid nitrogen–cooled CCD cameras, photon‐counting CCD cameras, and image‐intensifying CCD cameras (Castaño et al., 1996; Kennedy et al., 1997; Maire et al., 2000; Masamizu et al., 2006; Takasuka et al., 1998; White et al., 1995). These imaging studies have revealed heterogeneous expression of genes among individual cells to the same stimulus and have also contributed to identification of synchrony of circadian oscillation of clock gene expression in single cells (Welsh et al., 2004). Recently, electron‐multiplying CCD (EM‐CCD) cameras, which have higher sensitivity and image quality than previous ultra‐low‐light imaging cameras, were commercially released and used for bioluminescence microscopy (Hoshino et al., 2007; Kwon et al., 2010; Suzuki et al., 2011). Concurrent with the improvement in image sensor devices of ultra‐low‐light imaging cameras, we customized a short‐focal‐length imaging lens for bioluminescence microscopy and performed bioluminescence imaging of single live cells expressing the luciferase gene using a conventional CCD camera (Ogoh et al., 2014; Suzuki et al., 2007). This bioluminescence microscope is equipped with a conventional or EM‐CCD camera and has been widely used for gene expression analysis in chronobiology (Akashi et al., 2008; Dibner et al., 2008; Fukuda et al., 2011; Sato et al., 2006; Ukai et al., 2007; Yagita et al., 2010), neurobiology (Asai et al., 2008), developmental biology (Akiyoshi et al., 2014), medical research (Horibe et al., 2014; Sramek et al., 2011), signal transduction analysis (Hall et al., 2012; Roger et al., 2008; Sugiyama et al., 2014), molecular interaction analysis (Binkowski et al., 2009; Cosby, 2009), and radiation biology (Pratx et al., 2012, 2013).

In the case of analysis of transient gene expression at the single‐cell level with bioluminescence microscopy, identification of target cells transfected with the reporter gene using fluorescent markers before bioluminescence image acquisition will improve efficiency. Several hours are required for gene promoter assays. Establishing simultaneous fluorescence and bioluminescence microscopy will be useful for studying the heterogeneous response of gene expression in individual cells if a series of events from cellular signal transduction to gene expression regulated by specific transcription factors derived from the signaling cascade could be observed sequentially in the same single cells. Signal transduction and gene expression processes have only been imaged with fluorescence and bioluminescence microscopy as separate steps. Therefore, an imaging technique with fluorescence observation combined with bioluminescence microscopy is highly anticipated.

We previously tried to observe both processes in the same single cells using our bioluminescence microscope (Hatta‐Ohashi et al., 2009), which allows fluorescence observation with the transmittance method. We observed translocation of protein kinase C (PKC) fused to enhanced green fluorescent protein (EGFP) from the cytoplasm to the plasma membrane following stimulation with phorbol‐12‐myristate‐13‐acetate (PMA) and the binding activity of a transcription factor, nuclear factor of kappa light polypeptide B (NF‐κB). However, fluorescence emission of luciferin excited by the light used to induce EGFP fluorescence was extraordinarily strong, and we had to add luciferin to the culture medium after fluorescence observation.

In the present study, we optimized conditions for optical filter sets based on spectral properties of luciferin and the luciferin concentration based on the cell permeability of luciferin for fluorescence observation combined with bioluminescence microscopy, and we resolved the autofluorescence obstacle of luciferin during fluorescence observation. Using these optimized conditions, we present an example of sequential fluorescence and bioluminescence microscopy from signal transduction (PKCα translocation from the cytoplasm to the plasma membrane) to gene expression activated by NF‐κB in the same single cells.

## MATERIALS AND METHODS

### Luciferin Spectra

Beetle d‐luciferin, potassium salt (Promega, Madison, WI), was dissolved in Dulbecco's phosphate‐buffered saline (PBS (−); Nissui, Tokyo, Japan), pH 7.2, at a concentration from 0.1 to 1,000 μM, and its excitation and emission spectra were determined with a fluorescence spectrometer (F‐2500, Hitachi, Tokyo, Japan). The slit width for excitation and emission was 2.5 nm, the photomultiplier tube (PMT) gain was 700 V, and the scanning speed was 300 nm/min.

EGFP (GE Healthcare Science, Buckinghamshire, UK) containing a polyhistidine tag at the N‐terminus was expressed in *Escherichia coli* strain JM109 (DE3) (Promega) with the pRSET‐B expression system (Invitrogen, Carlsbad, CA) and was purified using a Ni‐NTA agarose resin column (Qiagen, Hilden, Germany). Excitation and emission spectra of EGFP were determined in citrate‐phosphate buffer, pH 7.0, using the same procedure as for the luciferin spectra.

### Plasmid Construction and Cell Culture

*EGFP* was inserted into a mammalian expression vector, pCDNA 3.1 (Invitrogen). The vector was transfected into HeLa cells (ECACC, Salisbury, UK) using the transfection reagent FuGene HD (Roche, Basel, Switzerland). The cells were cultured in 2 mL FluoroBrite DMEM (Life Technologies, Carlsbad, CA) containing 0 to 2 mM luciferin in 35‐mm glass‐bottomed dishes and were used for fluorescence microscopy.

Mouse *PKCα* was cloned with polymerase chain reaction (PCR) using a primer set (forward primer: 5′‐AAACTCGAGATGGCTGACGTTTACCCGGCCAAC‐3′, reverse primer: 5′‐CCCGGTACCTACTGCACTTTGCAAGATTGGGTG‐3′) derived from NCBI Reference Sequence NM_011101 from a mouse brain cDNA library (Takara Bio, Shiga, Japan) and was inserted in‐frame into the *Xho* I/*Kpn* I multiple cloning sites of vector pEGFP‐N3 (GE Healthcare Science) at the 5′‐end of *EGFP*.

Finally, a PKCα‐EGFP and luciferase co‐expression vector was constructed based on vector pBudCE4.1 (Invitrogen). *PKCα* fused to *EGFP* in pEGFP‐N3 was amplified with PCR using a primer set containing homologous sequences of pBudCE4.1 upstream from the *Pst* I site and downstream from the *Xba* I site (forward primer: 5′‐TCACTATAGGGAGACCCAAGCTTGTAATGGCTGACGTTTACCCGGCCAAC‐3′, reverse primer: 5′‐CTTCTGAGATGAGTTTTTGTTCGGATCCTTACTTGTACAGCTCGTCCATGC‐3′). This PCR product and the pBudCE4.1 digested with *Pst* I and *Xba* I were subjected to homologous recombination using the GeneArt Seamless Cloning and Assembly kit (Invitrogen). This yielded a vector constitutively expressing PKCα‐EGFP under control of the cytomegalovirus (CMV) promoter in the pBudCE4.1vector.

The region including a cis‐acting enhancer element sequence of NF‐κB to the TATA box promoter of the pNF‐κB(1)‐Luc TransLucent reporter vector (Panomics, Santa Clara, CA) at *Nhe* I/*Hind* III sites was removed and inserted into the pGL4.14 *Luc2* luciferase reporter vector (Promega). Then, the enhancer‐promoter‐*Luc2* region was removed, and the elongation factor 1α promoter region of pBudCE4.1 containing *PKCα* fused to *EGFP* constructed above was replaced with the enhancer‐promoter‐*Luc2* region at the *Nhe* I/*Kpn* I sites. Furthermore, the poly(A) signal/transcriptional pause site from pGL4.14 was added prior to the NF‐κB enhancer sequence using the *Nhe* I site for background reduction. Thus, the co‐expression vector that contained *PKCα‐EGFP* driven by the CMV promoter and *Luc2* driven by the TATA box promoter under control of the NF‐κB enhancer was constructed and transfected into HeLa cells as described above, and the cells were subjected to fluorescence and bioluminescence microscopy.

Vectors in which *EGFP* was replaced with *enhanced yellow fluorescent protein* (*EYFP*) or *red fluorescent protein* (*RFP, mKate2*, Evrogen, Moscow, Russia) were also constructed using the same procedure.

### Fluorescence and Bioluminescence Microscopy

**Figure 1:**
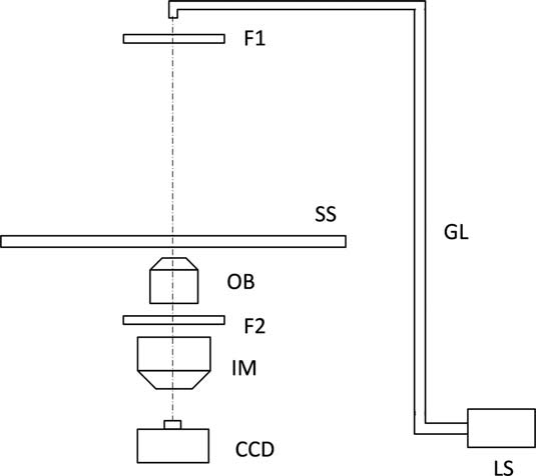
Diagram of the inverted bioluminescence microscope used in this study. A short‐focal‐length imaging lens (*f* = 36 mm for 0.2× or 90 mm for 0.5×) was used to capture dim bioluminescence image in this system. However, the shorter focal length of imaging lens leads to vignette of image on the light pass between objective and imaging lenses. To avoid this matter, the distance between the objective and imaging lenses was restricted mechanically to 17 mm. LS, light source (halogen lamp); F1, excitation filter; F2, emission filter; GL, glass fiber; OB, objective lens; IM, imaging lens; CCD, CCD camera; SS, sample stage.

Figure 1 shows a diagram of the inverted bioluminescence microscope used in our study (Luminoview LV200; Olympus, Tokyo, Japan). This microscope allows fluorescence observation with the transmittance method. A halogen lamp (LS) was used as both the source of transmitted bright‐field light and excitation for fluorescence observations. The light was directed into an excitation filter (F1) through a condenser lens with a glass fiber (GL). The emission filter (F2) was inserted between the objective (OB) and imaging (IM) lenses. A short‐focal‐length imaging lens (*f* = 36 mm for 0.2× or 90 mm for 0.5×) was used to capture dim bioluminescence images in this system (Ogoh et al., 2014; Suzuki et al., 2007). However, the shorter focal length of imaging lens leads to vignette of image on the light pass between objective and imaging lenses. To avoid this matter, the distance between the objective and imaging lenses was restricted mechanically to 17 mm. Therefore, a dichroic mirror unit could not be inserted between the objective and imaging lenses for fluorescence observation. The excitation and emission filters used were set 1 (BP460‐480HQ, Olympus and FF01‐550/49‐25, Semrock, New York, NY), set 2 (BP490‐500YFP, Olympus and FF01‐550/49‐25) and set 3 (FF01‐500/10‐25, Semrock and FF01‐550/49‐25) for EGFP or EYFP observation. We used set 4 (BP545‐580, Olympus and 610ALP, Omega, Brattleboro, VT) for mKate2.

The objective lens used was a UPLFLN100XO2PH (Olympus). The imaging lens was a 0.5× lens for filter selection experiments (Fig. 3) and a 0.2× lens for imaging of PCKα translocation to NF‐κB enhancer activation (Fig. 5). An EM‐CCD camera (ImagEM C9100‐13, Hamamatsu Photonics, Shizuoka, Japan) was used for capturing images. Details of conditions for capturing images (gain of EM‐CCD camera, exposure time, and time‐lapse imaging conditions) are described in Results and Discussion and in the figure legends.

### Luciferin Concentration Inside Cells

The concentration of luciferin inside and outside of HeLa cells was determined with a confocal laser scanning microscope (FV1000, Olympus). Confocal fluorescence images of HeLa cells cultured in PBS containing 25 mM HEPES and 0 to 3 mM luciferin were captured on 35‐mm glass‐bottomed dishes with the following conditions: objective lens, UPFLN60X (Olympus); excitation, 458 nm with a Multi‐Ar laser; laser power, 700 V; emission filter, BA505‐525 (Olympus); scan speed, 40 μs/pixel; pixel size, 0.138 μm. Arbitrary square regions were assigned inside and outside of cells, and average fluorescence intensity of the regions was plotted against luciferin concentration in culture medium. Luciferin concentration inside the cells was estimated by comparing the fluorescence intensity outside the cells and the luciferin concentration in culture medium.

### Kinetic Constant of Luciferase

The Michaelis‐Menten constant (*K*_m_) value of luciferase for luciferin was determined from the luminescence intensity with a luminometer (Luminescencer JNR II, Atto, Tokyo, Japan). The luciferase gene (*Luc+*) from the pGL3‐control vector (Promega) was inserted into pRSET‐B and expressed in *Escherichia coli* strain JM109 (DE3), and the luciferase was partially purified with a Ni‐NTA agarose resin column. Luminescence intensity was determined with the luminometer in 50 mM Tris‐HCl (pH 8.0) containing 50 μg/mL of partially purified luciferase (enough for *K*_m_ determination), 2 mM ATP, 4 mM MgSO_4_, and luciferin (2.5–320 μM). Time course of light emission was measured for 10 s with 0.02 s gated time, and the reaction rate was determined by peak light intensity. The *K*_m_ value was estimated by curve fitting against the Michaelis‐Menten equation using the least squares method. The amino acid sequences of Luc+ and Luc2 are identical, although the nucleotide sequence of *Luc2* was modified for mammalian expression (GenBank Accession Numbers U47296 and AY864928).

## RESULTS AND DISCUSSION

### Luciferin Spectrum

**Figure 2:**
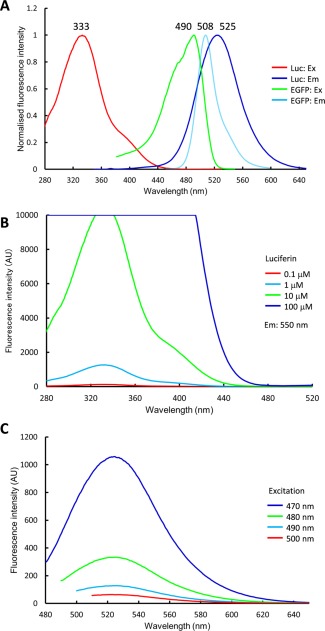
Spectral properties of luciferin and EGFP. **A**: Normalized excitation and emission spectra of luciferin and EGFP. Numbers above the curves indicate the peak wavelengths (in nm). Ex, excitation; Em, emission. **B**: Excitation spectra of luciferin (0.1–100 µM) with fluorescence measured at 550 nm. **C**: Emission spectra of 1 mM luciferin scanning the excitation range 470–500 nm. [Color figure can be viewed in the online issue, which is available at wileyonlinelibrary.com.]

**Figure 3:**
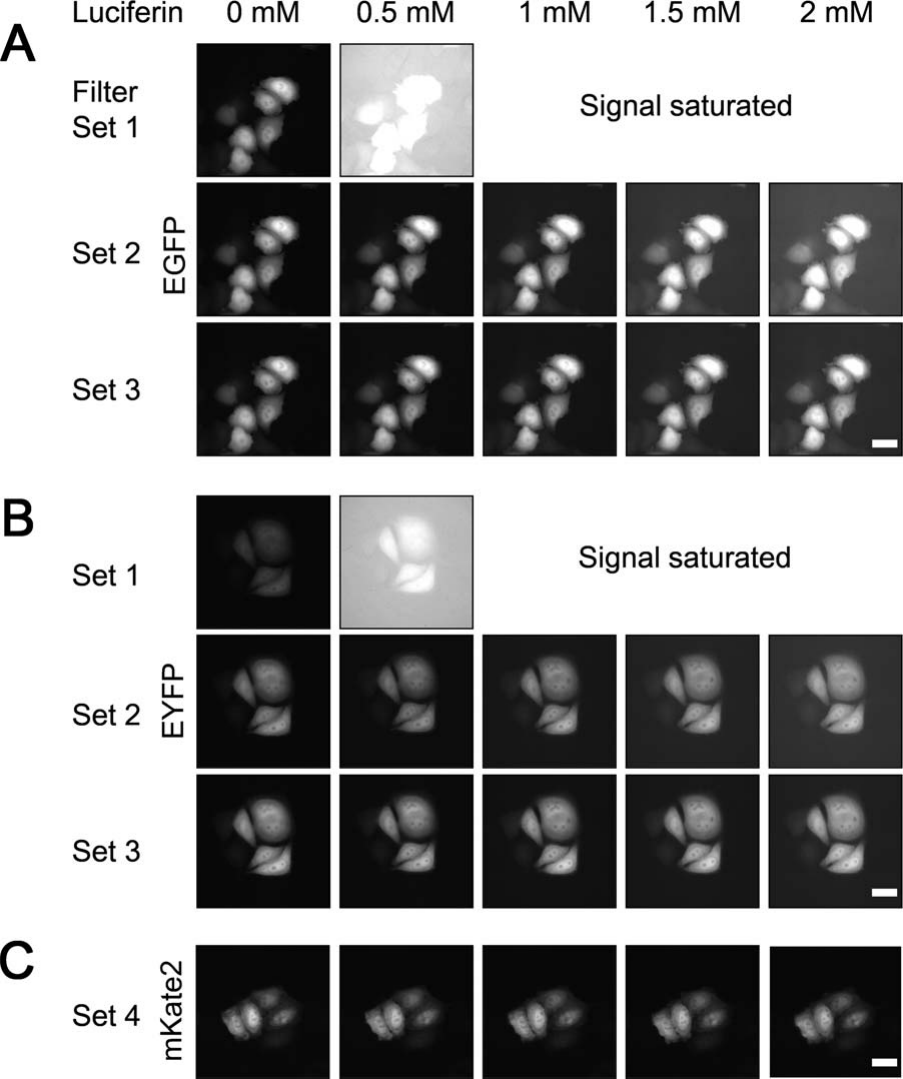
Fluorescence images of HeLa cells expressing EGFP, EYFP, or mKate2. The luciferin concentration in the culture medium ranged from 0 to 2 mM. **A**: EGFP images captured by filter set 1, 2, or 3. EM‐CCD gain, 410; exposure time, 150 ms. **B**: EYFP images captured with filter set 1, 2, or 3. EM‐CCD gain, 208; exposure time, 150 ms. **C**: mKate2 images captured with filter set 4. EM‐CCD gain, 700; exposure time; 150 ms. All images are shown with no level adjustment. Filter set 1, BP460‐480HQ (Ex) and FF01 550/49‐25 (Em); set 2, BP490‐500YFP (Ex) and FF01 550/49‐25 (Em); set 3, FF01 500/10‐25 (Ex) and FF01 550/49‐25 (Em); set 4, BP545‐580 (Ex) and 610ALP (Em). Ex, excitation; Em, emission; Scale bar, 20 μm.

Figure 2A shows the normalized excitation and emission spectra for luciferin and EGFP. The peak wavelengths of the excitation and emission spectra for luciferin were 333 and 525 nm and for EGFP were 490 and 508 nm, respectively. Because the range of half width (463–479 nm) of the EGFP excitation filter (BP460‐480HQ) did not overlap with the tail of the luciferin excitation spectrum at ∼450 nm, we reasoned that fluorescence observation of cells expressing EGFP was possible in culture medium containing luciferin using the excitation and emission filters BP460‐480HQ and FF01‐550/49‐25 (half width: 524–578 nm). However, fluorescence observation was inhibited by the strong fluorescence background of luciferin that was excited by the light used to induce EGFP fluorescence (Fig. 3A).

Generally, we found that a luciferin concentration of ∼1 mM was required for the gene promoter assay, and this concentration was much higher than that required for fluorescence spectrometry. Therefore, a high concentration of luciferin affected tail elevation of the excitation spectrum at ∼450 nm. Figure 2B shows the excitation spectra of luciferin at 0.1, 1, 10, and 100 μM. The tail of the spectra at ∼450 nm rose in accordance with the luciferin concentration. Figure 2C shows the emission spectra of 1 mM luciferin excited by light at 470, 480, 490, and 500 nm. The fluorescence emission of luciferin decreased to 5.9% at a peak height of 525 nm in proportion to the increase in the excitation wavelength from 470 to 500 nm. Thus, the optimal excitation wavelength was ∼500 nm.

### Fluorescence Images of Cells in the Presence of Luciferin

Figure 3 shows fluorescence images of HeLa cells expressing EGFP (panel A) and EYFP (panel B) captured with an LV200 microscope. The excitation filter was BP460‐480HQ (463–479 nm), BP490‐500YFP (490–501 nm), or FF01‐500/10‐25 (492–506 nm), and the emission filter was FF01‐550/49‐25 (524–578 nm). Values in parentheses are the range of the half width of the filters. Luciferin concentration in the culture medium ranged from 0 to 2 mM. For EGFP fluorescence (Fig. 3A), the gain of the EM‐CCD camera was 410 and the exposure time was 150 ms. For excitation with filter set 1 (BP460‐480HQ), the background of the image increased beginning with 0.5 mM luciferin, and images were saturated at >1 mM luciferin. On the other hand, EGFP images could be obtained with set 2 (BP490‐500YFP) and set 3 (FF01‐500/10‐25) excitation in the range of luciferin concentration examined (0.5–2 mM), although the background of the image increased slightly in accordance with the luciferin concentration. The background of images captured with set 3 excitation was slightly lower than with set 2 excitation.

For EYFP fluorescence (Fig. 3B), to capture the same signal intensity as with EGFP imaging at 0 mM luciferin using set 2 excitation, the gain of the EM‐CCD camera could be reduced from 410 to 208 with the same exposure time. These images were essentially the same as those obtained with EGFP, but the background was lower. Therefore, set 3 excitation was deemed suitable for EGFP or EYFP imaging of cells in culture medium containing luciferin, and EYFP was much better than EGFP for fluorescence observation with a filter set.

Although the luciferin concentration affected the background fluorescence of images, EGFP and EYFP images of cells could be obtained with 2 mM luciferin and set 2 or 3 excitation. Because fluorescent protein expression under control of the CMV promoter is strong, the signal‐to‐background ratio of the images was acceptable. However, as the expression level of a fusion fluorescent protein or with conventional promoters is not very strong, adjustment of the capture conditions (gain of CCD camera, exposure time, power of excitation) is generally required to reduce the background fluorescence. Therefore, a lower concentration of luciferin is much better for fluorescence imaging.

Figure 3C shows fluorescence images of HeLa cells expressing RFP (mKate2). In this case, no fluorescence background from luciferin was seen. Thus, EGFP, EYFP, and RFP can be used as fluorescent markers.

### Luciferin Concentration inside Cells

**Figure 4:**
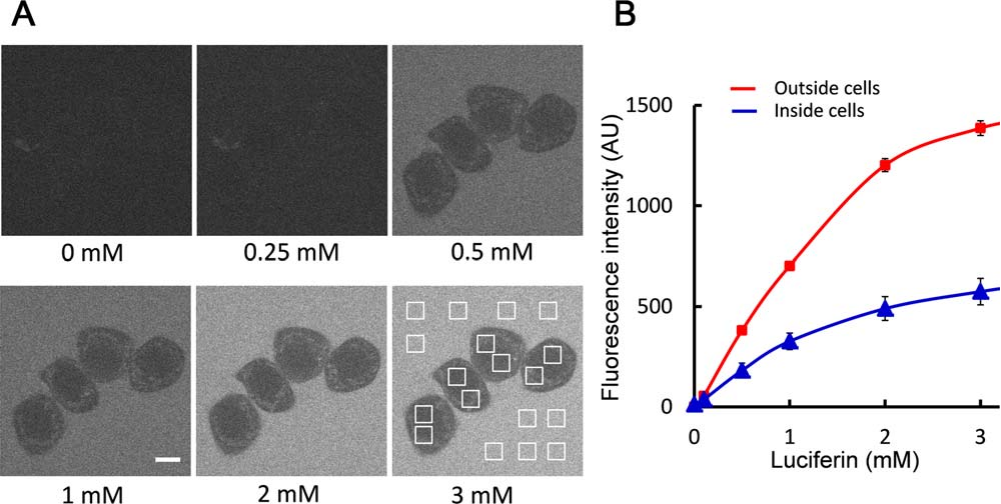
Estimation of the luciferin concentration in cells. **A**: Confocal fluorescence images of HeLa cells cultured in PBS containing 25 mM HEPES and luciferin ranging from 0 to 3 mM. Eight and 10 arbitrary square regions were assigned inside and outside the cells, respectively. Scale bar; 10 μm. **B**: Average fluorescence intensities of the assigned regions inside and outside the cells were plotted against luciferin concentration in culture medium. The data points and error bars shown on the graph are the mean ± SD. [Color figure can be viewed in the online issue, which is available at wileyonlinelibrary.com.]

Figure 4A shows confocal fluorescence images of HeLa cells cultured in PBS containing 25 mM HEPES and luciferin from 0 to 3 mM. Eight and ten arbitrary square regions were assigned inside and outside cells, respectively, and the average fluorescence intensity of the regions was plotted against luciferin concentration in culture medium (Fig. 4B). Fluorescence quantum yield of luciferin shows increase in alkaline condition (p*K*_a_ = 8.25) (Seliger et al., 1961). However, pH value between inside and outside cells is considered to be same in physiological culture condition. Therefore, we assumed that fluorescence quantum yield of luciferin was same between inside and outside cells. As shown in the graph, the luciferin concentration inside the cells was approximately half the concentration outside the cells. Meanwhile, luciferin transporter proteins (ATP binding cassette transporter protein, multidrug resistance protein, organic anion transporter protein) and their inhibitors were reported (Huang et al., 2011; Patrik et al., 2014; Zhang et al., 2007). It is considered that the concentration gradient of luciferin between inside and outside of cells is caused by not only simple diffusion but also the transporter proteins.

Because luciferin is used as a substrate of the luciferase reporter enzyme, a luciferin concentration at least twice the *K*_m_ value of the luciferin‐luciferase interaction is required inside cells. The *K*_m_ value of the luciferin‐luciferase interaction was estimated as 15.7 μM with our curve fitting method. Therefore, at least 32 μM luciferin is required (64 μM in culture medium) for live‐cell reporter analysis, and we concluded that luciferin higher than 100 μM in culture medium is sufficient for fluorescence observation combined with bioluminescence reporter analysis.

### PKCα Translocation and Gene Expression Activated by NF‐κB

**Figure 5:**
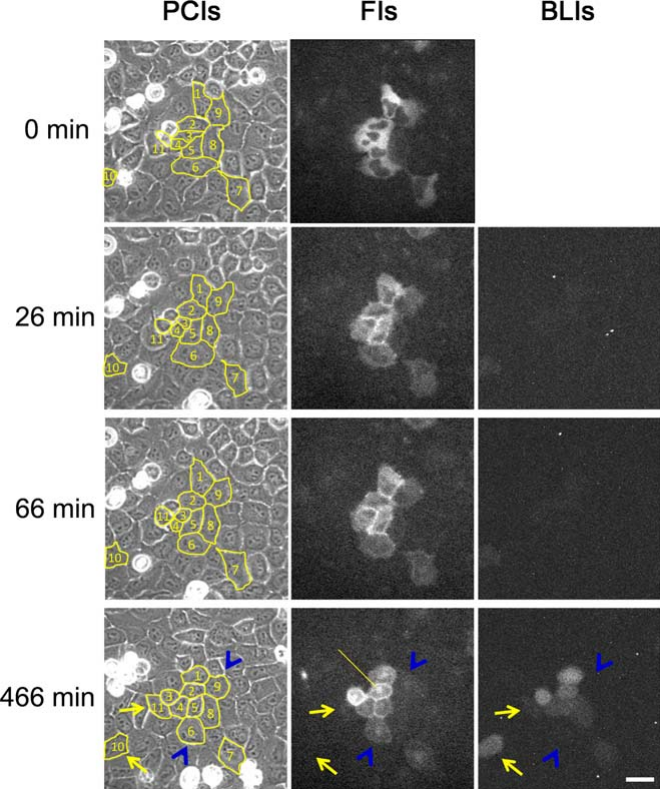
Fluorescence images (FIs) of HeLa cells expressing PKCα fused to EYFP, and bioluminescence images (BLIs) of gene expression activated by NF‐κB together with phase‐contrast images (PCIs) of the same cells. Images displayed are from 0 to 466 min after stimulation with PMA (30 ng/mL). The luciferin concentration in the culture medium was 0.2 mM. The EM‐CCD gain and exposure time were 1,200 and 300 ms for FIs, and 1,200 and 10 min for BLIs, respectively. The filter set for FIs was set 3 (FF01 500/10‐25 and FF01 550/49‐25) as in Figure 3. Cells that responded to PMA were numbered and traced with a yellow line on PCIs to track migration. The line indicates PKCα localization in the Golgi. Arrows indicate cells that showed gene expression activity but no PKCα translocation. Arrowheads indicate cells that showed no gene expression activity but did show PKCα translocation. Scale bar, 20 μm. [Color figure can be viewed in the online issue, which is available at wileyonlinelibrary.com.]

Figure 5 shows fluorescence images of PKCα fused to EYFP and bioluminescence images of gene expression activated by NF‐κB, together with phase‐contrast images of the same cells. The EM‐CCD gain and exposure time were 1,200 and 300 ms for fluorescence images and 1,200 and 10 min for bioluminescence images, respectively. Fluorescence images were captured for 22 min with a 4‐min interval, and then fluorescence images and bioluminescence images were captured sequentially for 13.5 h with a 20‐min interval. The luciferin concentration in the culture medium was 0.2 mM. Cells that responded to stimulation with PMA (30 ng/mL) were numbered (No. 1–11) and traced with a yellow line on the phase‐contrast images to track migration. At the beginning of PMA stimulation, PKCα was homogeneously distributed throughout the cytoplasm in nine cells (No. 1–9). Then, PKCα localized to the plasma membrane from 6 to 26 min (40 cells in three experiments) after stimulation. From 10 to 146 min after stimulation, PKCα also appeared in endomembranous structures (29 cells in three experiments, line in Fig. 5), which are considered to be the Golgi (Aschrafi et al., 2003). On the other hand, nine cells (No. 1–11 except 6 and 9) showed NF‐κB–induced gene expression activity, which reached a maximum from 90 to 780 min after stimulation and then decreased (26 cells in three experiments). However, some cells that showed gene expression activity were not the same as those that showed PKCα translocation. The arrows indicate cells (No. 10 and 11) that showed gene expression activity but no PKCα translocation. On the contrary, the arrowheads indicate cells (No. 6 and 9) with no gene expression activity but with PKCα translocation. Thus, at the single‐cell level, the gene expression response was not entirely concordant with upstream signaling following a single stimulation with PMA.

Using western blotting and a luciferase reporter assay, Shin et al. (2007) showed that PKCα acts as an upstream regulator of NF‐κB in PMA‐mediated induction of matrix metalloproteinase‐9 in lung epithelial cells. We traced the pathway from the PKC‐dependent signal transduction cascade to gene expression activated by NF‐κB using fluorescence and bioluminescence microscopy. This demonstrational experiment enabled observation of a heterogeneous response from signal transduction to gene expression in the same cells (arrows and arrowheads in Fig. 5). Recently, single‐cell transcriptome analysis of stem cells was developed in vitro, and single‐cell heterogeneity analysis is considered important for determining stem cell function and the roles they play in development (Itzkovitz and van Oudenaarden, 2011; Schroeder 2011; Tang et al., 2011). We believe that our technique is a powerful imaging tool for analysis of heterogeneous gene expression together with upstream signaling in live single cells.
